# Awareness Level of Business Students regarding Drinking Water Safety and Associated Adulteration Accidents: A Multinomial Logistic Regression Approach

**DOI:** 10.1155/2022/7492409

**Published:** 2022-08-29

**Authors:** R. M. Ammar Zahid, Muzammil Khurshid, Wajid Khan, Ziyue Hong, Hawa Kasule

**Affiliations:** ^1^School of Accounting, Yunnan Technology and Business University, Kunming, China; ^2^Department of Banking and Finance, University of the Punjab, Gujranwala Campus, Gujranwala, Pakistan; ^**3**^ Department of Business Management, University of Baltistan, Skardu, Pakistan; ^4^Department of Linguistics, English Language Studies, and Communication Skills, Makerere University, Kampala, Uganda

## Abstract

The industrialization of metropolis urban areas with dry and steppe climates raise substantial environmental contamination, particularly in the water domain. This research investigated the awareness levels of business students toward drinking water quality and safety. We further explored the knowledge of the business students regarding drinking water issues and remedies. Eighty-four percent of respondents were happy with the quality of their drinking water, according to the findings. Approximately 66% of respondents paid special or rather high attention to drinking water quality and contamination incidents, particularly regarding possible harm to the human body and health, impact scope, and accident reasons. Few respondents reported to the health department or phoned the water safety department; 47.5% of respondents resolved drinking water issues independently. Age and education level did not play a significant role in the degree of public satisfaction with water quality or the public's perception of water pollution incidents; however, business students in Samundri were more satisfied with their drinking water quality, and residents of Faisalabad Sadar were more aware of drinking water contamination incidents than residents in areas without such a network. Respondents with higher levels of education were more aware of water quality and pollution incidents than those with lower levels of education. The steppe climate, diverse human activities, and industrialization led to water pollution. The current research findings may provide fundamental data for efficient water management in the most populated and industrialized regions.

## 1. Introduction

A critical global issue with increased urbanization and industrialization is the availability of safe drinking water. Access to clean water is a significant health and development concern at the national, regional, and local levels [1]. Local government and schools have learned that the availability of clean drinking water is not sufficient; general population plays an essential role in water management [2]. Public acceptability of drinking water is also included in the World Health Organization's drinking water quality recommendations [1]. Therefore, popular opinions about drinking water safety and contamination incidents cannot be overlooked [3]. Awareness of environmental concerns is crucial to the effectiveness of public environmental engagement [4]. Numerous studies have demonstrated that boosting ecological awareness and understanding among the general population is vital to the effectiveness of pollution control [5, 6]. The public's knowledge of safe drinking water is pertinent to promoting home water treatment, selecting drinking water sources by households, and avoiding water contamination incidents [7].

Established water supplies cause inadequacy and deterioration of fresh water, resulting in severe water scarcity [8, 9]. Pakistan is the seventh-most water-scarce area in the world. Most emerging nations, including Pakistan, India, Africa, and Bangladesh, are progressively using water with deteriorating quality owing to human activities [10, 11]. In southern Asia, Pakistan has dry to semiarid climates in various regions. Due to urbanization, the massive population faces several water-related issues [12–14]. In Pakistan, water availability is steadily declining; by 2025 and 2050, it will fall to alarmingly low levels of 660 and 575 ft^3^ and 877 m^3^/year, respectively. The current study focused on third big city of Pakistan, Faisalabad is regarded as a polluted industrial (textile, ice, pharmaceutical, wheat, cotton, sugar, and food) city due to inadequate treatment facilities and the fact that more than 90% of samples exceeded WHO guidelines for K, Na, Cl, total dissolved solids (TDS), and SO_4_ [15, 16].

Awareness of drinking water safety and quality has been studied by researchers in different countries. For instance, Mahler, et al. [17] evaluated the drinking water issues and concerns of the urban public in the United States and discovered that the urban populace is satisfied that their home drinking water is safe. In Austria, 75% of survey respondents were entirely happy with the quality of drinking water, according to research by Fröhler and Elmadfa [18]. In China, Wang, et al. [19] surveyed public awareness about water safety in two rural counties of Henan province. This research demonstrated the significance of quality perception, service satisfaction, and water source selection in assessing public knowledge of drinking water safety and accident risk. A greater understanding of the elements that impact public awareness of drinking water may enhance water management, consumer services, and preventing and controlling water pollution accidents. Numerous variables have been identified as influencing general knowledge of drinking water quality. Water sources, water treatment methods, and water distribution networks may readily impact the quality and safety of drinking water [1]. The link between water quality and people's livelihood is tight, and access to clean drinking water is crucial for health [1]. Awareness of water quality and risk resulted from a complex interaction of multiple factors, including water taste, odor, clarity, socioeconomic characteristics, demographic characteristics, water treatment, geographic location in the distribution system, and information provided by local media [2, 7, 20–22]. For operational drinking water delivery systems, the amount of water, water pressures, and failures may also impact the quality of drinking water [23]. However, recent drinking water safety awareness studies have focused mostly on bottled water usage, municipal water, and recycled water [19]. There is research on the awareness level of business students regarding drinking water safety and associated adulteration accidents by using the multinomial logistic regression approach. Consequently, the purpose of this research was to explore the facts of general knowledge of drinking water safety and water pollution accidents in Faisalabad city, Punjab Province, Pakistan, as well as to analyze information regarding public awareness and attitudes toward drinking water and water pollution. This research will contribute to avoiding drinking water pollution and enhancing water management, particularly from the standpoint of public engagement with empirical evidence from a semiarid industrialized metropolitan city.

## 2. Materials and Methods

### 2.1. Studied Area Profile

According to the 2017 census, Pakistan has a total population of 207.68 million (106.3 million males and 101.3 million females). Punjab is the most pulpous province in the country (area 205,345 km^2^; population 109,989,655). With a growing population, Faisalabad is considered the second megalopolis in Punjab (area of 5,857 km^2^; population 7,882,444), with a growth rate of 1.98% [24]. The annual rainfall was measured at 408 mm. The maximum recorded temperature was 45°C, while the wind speed was 94 mph [25]. Today, Faisalabad is a thriving industrial center with several textile, dye, fertilizer, industrial chemical, pulp and paper, printing, industrial products, and agricultural equipment manufacturers, among others [26]. Most industrial wastewaters were dumped untreated into the two main drains, Paharang and Madhuana. Both the Paharang and the Madhuana drains are administered by the Irrigation Department. The Paharang drain ultimately empties into the Chenab River, while the Madhuana drain empties into the Ravi River. Faisalabad's oxidation ponds consisted of anaerobic and facultative ponds. In its vicinity, untreated wastewater has been utilized for 50 years to cultivate crops, vegetables, and fodder [27].

### 2.2. Research Design and Measurement

**Figure 1:**
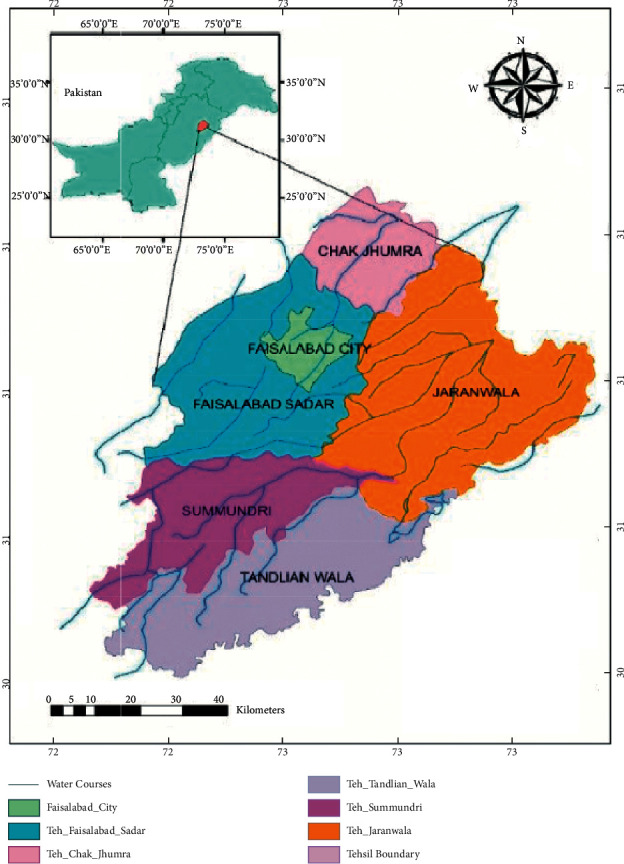
The map of study areas (source: Sohail, et al. [28]).

The objective of current research is to empirically investigate the attitude of business students toward water safety and pollution accidents. For this purpose, a descriptive and correlational cross-sectional, questionnaire-based survey was conducted among the business students of five tehsils (i.e., Chak Jhumra, Faisalabad Sadar, Jaranwala, Samundri, and Tandlianwala) of the Faisalabad district, Pakistan.  Figure 1 is a map of the research region. It will provide a valuable reference for drinking water control and prevention in other city areas of Pakistan as important because the results could provide a useful reference for drinking water control and other developing countries.

We adopted the measures of awareness about water safety and contamination accidents based on prior studies [18, 19]. The comprehensive questionnaire contains 15 items (5 demographic questions, 6 for drinking water safety, and 4 for water pollution accidents); each questionnaire only takes about 5 min to finish. The first 5 items measure the demographic characteristics of the respondents, such as name (optional), age, gender, education, and tehsil of residents. The next part of the questionnaire measures public awareness about drinking water safety (consisting of 6 questions). The awareness about the “main source of drinking water” is measured with a close-ended question with 5 items, that is, (1) tap water, (2) barreled or bottled water, (3) well water, (4) spring water, and (5) others. Public attention level to local drinking water quality is measured on the 4 items on the Likert scale, that is, (1) special attention, (2) comparatively high attention, (3) not concerned, and (4) no answer. The public's satisfaction level concerning drinking water quality is also measured by 4 items on a Likert scale, that is, (1) very satisfied, (2) relatively satisfied, (3) dissatisfied, and (4) no answer. Five items on a Likert scale measure public trust level in the safety of drinking water, that is, (1) confident, (2) relatively confident, (3) somewhat worried, (4) extremely worried, and (5) no answer. Public awareness about the problems with tap water quality is measured by 4 items, that is, (1) never had problems, (2) had problems once or twice a year, (3) had problems frequently, and (4) no answer. Actions taken to solve problems that arise with tap water and solve problems are measured by 5 items, that is, (1) solve problems by themselves, (2) help by local water utility, (3) complain to the local department of health, (4) help by the residential property maintenance staff, and (5) call the local government telephone hotline for help.

The last section of the questionnaire measures public awareness about the drinking water contamination accidents (consisting of four questions). Attention to the water pollution events is measured by four-item Likert scale, that is, (1) pay special attention, (2) follow in free time, (3) not concerned, and (4) no answer. Five items measure attention to the specific water pollution events, that is, (1) damage to human health, (2) influence scales, (3) cause of accident, (4) accident information publication, and (5) accident treatment procedures. Emergency response provider in water contamination accidents is measured by five items, that is, (1) health department, (2) environmental protection department, (3) water resources department, (4) propaganda department, and (5) housing and urban, rural development department. Things to reduce pollution emergencies are measured by four items, that is, (1) strengthening supervision, (2) resource management, (3) propaganda for protecting the knowledge, and (4) increasing the intensity of the punishment.

All the constructs (observable and latent) of the questionnaire were initially constructed in English. Later, the questionnaire was translated into Urdu (the national language; the questionnaire is given in supplementary material (available here). The survey was conducted among the residents of five tehsils of Faisalabad, Pakistan. To eliminate any translation discrepancies, the questionnaire was translated into Urdu and reviewed by two academic experts fluent in English and Urdu.

### 2.3. Sampling and Data Collection

After modifying and incorporating experts' suggestions, the survey instruments have been given to the final year students of management sciences departments of two renowned Pakistan universities in Pakistan for pilot testing, about 50 in total. Overall findings of pilot testing inveterate the reliability of the questionnaire scale. Finally, following the sampling method used by other researchers [28, 29], the questionnaire survey was conducted among the residents of 5 tehsils of Faisalabad with the help of final year students (100 questionnaires distributed in each tehsil). The objective of using purposive sampling was to balance respondents from each tehsil of Faisalabad.

We adopted the anonymous filling-in method to keep confidentiality and integrity (the first question about names was optional). Furthermore, the information provided by the respondents is only used for research purposes, and personal information will not be disclosed. It was optional for them to respond; if any resident refused to participate, a replacement was provided. A total of 500 questionnaires were distributed in 5 tehsils of District Faisalabad, Pakistan. In total, 408 questionnaires were returned (83.8% response rate); 84, 57, 108, 54, and 105 responses were received, respectively, from Chak Jhumra, Faisalabad Sadar, Jaranwala, Samundri, and Tandlianwala tehsils of Pakistan. Questionnaire responses with missing values and unengaged data were removed. The final data set used for analysis consists of 399 responses in total. Survey data were gathered, coded, and put into Microsoft Excel. Then, using StataMP econometric software, the statistical analysis of data was conducted.

### 2.4. The Statistical Approach

The awareness about drinking water safety and contamination accidents is analyzed through frequency distribution tables and a multinomial regression model. In particular, using discrete choice regression models is appropriate to investigate the factors influencing public awareness about water safety and adulteration issue. Because ordinary least squares (OLS) are not an appropriate statistical methodology for binary data, logistic or probit estimation methods are used [30]. Furthermore, our dependent variables are tetrachotomous/polychotomous, so the multinomial logistic regression (MLR) model is applied. *Y* represents the dependent variable, with four choices, that is, *k* = 4. Then, the multinomial regression consists of a set of four logistic models that, after being normalized for the reference category of *Y*, allow us to calculate the probability of *Y* taking the value of each of the three categories. The probability of *Y* is given by the matrix notation equation as follows:

(1) $$\begin{array}{c} P\left(Y=\frac{1}{X}\right)=\frac{1}{1+\sum_{k=2}^{3}e^{x\beta_{k}}},\;P\left(Y=\frac{2}{X}\right)=\frac{e^{x\beta_{2}}}{1+\sum_{k=2}^{3}e^{x\beta_{k}}},P\left(Y=\frac{3}{X}\right)=\frac{e^{x\beta_{3}}}{1+\sum_{k=2}^{3}e^{x\beta_{k}}},P\left(Y=\frac{4}{X}\right)=\frac{e^{x\beta_{4}}}{1+\sum_{k=2}^{3}e^{x\beta_{k}}}, \end{array}$$

where *X* is the matrix of independent variables and *β* is the vector of coefficients.

The logit of each nonreference category relative to the reference category is contingent upon a set of explanatory factors. The model is estimated using the approach of maximum likelihood.

## 3. Results and Discussion

### 3.1. Demographics of Respondents

Table 1 presents the demographic breakdown of the sample. Most respondents were male (52.9%), between the ages of 35–50 and 20–34, with bachelor's degrees. These findings were consistent with the gender, age, and education demographics of these five tehsils of Faisalabad.

### 3.2. Awareness about the Safety of Drinking Water

#### 3.2.1. Sources of Drinking Water

One of the key factors in the World Health Organization's Universal Health Coverage (UHC) program is the WASH (water, sanitation, and hygiene), and household water security has prime importance in it [31]. Panel A of Table 2 provides the survey results of residents' drinking water sources. Most respondents (37.2%) reported using tap water as their primary source of drinking water, followed by bottled water (33.6%). Very little spring and the well water were consumed. These are distinct from developed nations. In these developed cities, bottled or barreled water use has expanded dramatically during the previous decade [32]. Due to massive industrialization in the city, the water quality of surface water and groundwater is not good; therefore, the proportion of using bottled or barreled water is increasing with time. Thus, several local water filtration utilities have been developed at different locations of all five tehsils of Faisalabad, and bottled (filtered) water is becoming popular in these communities. Several studies have shown that when customers are unsatisfied with the municipally supplied tap water, they often shift to bottled or barreled water [19, 32].

#### 3.2.2. Attention of the Public to Local Drinking Water Quality

Panel 2 of Table 2 shows that 36.0% of respondents paid great attention to local water quality, 20.2% paid somewhat high attention, and 27.9% were unconcerned with local drinking water quality. The following technologies are examined from the water source through the distribution points: water sources and intakes, water-lifting devices, power technologies, water treatment, storage, and distribution. Each of these subsystems' technologies must perform effectively to maintain a dependable water supply and safe water quality [33]. A clean and accessible water supply is essential to public health and welfare [34]. Most water treatment facilities in all five tehsils of Faisalabad are modest, with mostly centralized (or sometimes decentralized) township (local Baldia) and rural water supplies and a water supply capacity of less than 1,000 m^3^/d. The water items and equipment are quite basic and rudimentary. There is no water treatment equipment in these purification facilities, so their purification ability is extremely restricted, and some impurities should not be eliminated. And, as a result of the fact that many people were keen to know whether their drinking water was clean or not, the majority of respondents paid close attention to its quality.

#### 3.2.3. Student Satisfaction with Drinking Water Quality

Panel 3 of Table 2 displays survey findings on students' satisfaction with drinking water quality. About 21.9% of respondents were very happy with the quality of their drinking water; 62.0% were somewhat content; and 11% were unsatisfied with the quality of their drinking water. Multiple studies [19, 29, 35] have examined consumer satisfaction with the quality of drinking water. Numerous variables, including availability and safety of water sources, taste, and attitudes toward chemicals, influence the quality of drinking water [2, 7, 28, 29]. Few inhabitants utilize barreled or bottled water, but they often believe that bottled water is a clean and safe product, which may explain the 100% satisfaction rate for barreled or bottled water. Users of tap water sources were only happy with the quality of their drinking water. Well water and spring water are sourced from decentralized water sources that are obtained directly from the water source, without or with little infrastructure. Dissatisfaction among respondents was mostly attributable to sensory qualities such as water turbidity, red color, and disagreeable flavor and odor. If locals fully understand the water treatment process and the significance of all water quality indicators, their level of satisfaction with the quality of their drinking water is likely to alter.

#### 3.2.4. Residents Trust in Drinking Water Safety

Panel 4 of Table 2 displays the results on the public confidence level in drinking water safety. In total, 28.5% of respondents felt confident in the safety of their drinking water, while 27.7% felt moderately confident. Water resources and quality have been recognized as the most significant components of public confidence in drinking water safety [36]. Around 33.6% of respondents said they were somewhat concerned about safety of their drinking water, while 7.3% were highly concerned. About 40% of citizens in these two counties lacked confidence, which may be attributable to the crude or basic water treatment used. In Faisalabad's rural regions, there are no standards governing the transmission of drinking water quality, and water treatment facilities seldom disclose the values of drinking water quality indicators. On occasion, residents acquire information on the quality of their drinking water through television, newspapers, or the Internet. According to research, little information and a few stories are insufficient to successfully alter public image [37]. Consequently, many inhabitants of the five tehsils of Faisalabad are concerned about the quality and safety of the local drinking water, and some locals are concerned about the safety of the drinking water.

#### 3.2.5. Knowledge regarding Common Tap Water Problems and Solutions

Students often have issues with the taste, flavor, smell, and look of their drinking water, such as water that is initially clear but generates brown, orange, reddish stains or sediment, or water that has a metallic taste. As indicated in panel 5 of Table 2, 24.1% of respondents claimed they had never had difficulties with the quality of their tap water; 35.0% reported having problems once or twice a year; 33.6% said regular tap water concerns; and 7.3% did not reply. Issues with drinking water include white froth, rust color, disagreeable odor, turbidity, red worms, and other contaminants. Water supply issues include water shortages and periodic water scarcity. However, there are many more issues with water quality that cannot be observed, and respondents do not completely comprehend the majority of water quality indicators of their drinking water. Hence, the majority of respondents claimed no or few concerns with tap water.

#### 3.2.6. Student Awareness to Solve Common Tap Water Problems and Solutions

Panel 6 of Table 2 also displays respondents' knowledge of solutions for tap water issues such as strange water quality, pipeline damage, faucet water leakage, and so on. Around 47.5% of residents repaired pipes and faucets themselves and filtered unclean water using a home water purifier; 24.8% relied on the local water utility; 8.8% of respondents complained to the local department of health; 9.5% sought assistance from the residential property maintenance staff; and only 9.5% of respondents called the local government telephone hotline for assistance. These data indicate that people often resolve difficulties with their drinking water on their own by contacting the local water provider for assistance. Rural regions have a dearth of knowledge about drinking water problems and remedies. Many households resolve water-related issues without assistance from water treatment facilities or monitoring offices. When homeowners turn to municipal water utilities or monitoring agencies for assistance, these departments cannot resolve the situation promptly. Some folks are completely unaware of how to contact these agencies.

### 3.3. Awareness about Water Pollution Events

#### 3.3.1. Awareness of Water Contamination Incidents among the Public

Panel 1 of Table 3 shows that 34.3% of respondents stated they pay close attention to news of water contamination incidents, while 17.5% indicated they are unconcerned about such incidents. About 43.3% indicated that they pay attention only in their free time. In recent years, severe water pollution incidents, such as contamination with heavy metals, algal blooms, organic chemical spills, and microbiological contamination, have caused public concern [25, 28, 29]. In response to the question, “What type of water pollution incident do you pay attention to?” some residents wanted to know whether long-term consumption of bottled or barreled water is harmful to the human body and what diseases could be caused by drinking unclean water for an extended time.

#### 3.3.2. General Understanding of Water Pollution Incidents

Panel 2 of Table 3 depicts the results of awareness of water contamination incidents. About 83.9% of respondents knew of the possible health risks associated with water contamination incidents. And 4.4% are aware of the influence scales of such accidents, and 6.6% of the respondents are mindful of the cause of such accidents. Accident information impact acquired from information in government publications on water contamination incidents and accident treatment methods.

#### 3.3.3. Emergency Response Provider and Preventative Measures for Water Pollution Incidents

Panels 3 and 4 of Table 3 show that 36% of residents believe that the health department is the emergency resource provider in an emergency. In comparison, 44.9% think the water resource department should be contacted in case of emergency. About 27% of respondents agreed with enhanced supervision and monitoring, and 42.7% says there should be better resource management to solve water contamination emergencies. About 21.3% of respondents said that improved awareness and education and higher fines for polluters might also contribute to reducing pollution accidents.

Faisalabad district government established water quality monitoring networks in each tehsil. Despite this, geographical coverage remains low owing to the country's vastness. Environmental campaigners in Pakistan assert that the Ministry of Environmental Protection's sanctions for pollution and illegal actions are insufficient in most situations [16, 28, 29]. Such regulations demonstrate government resolve but are inadequate to inspire terror. Some departments can oversee the water source and utilities to implement these laws. The department of health monitoring is empowered to lead water treatment facilities. When a water treatment facility is deficient, it should be penalized to enhance the water treatment technology and strengthen the building of the water distribution network to improve the water quality. The department of environmental protection is empowered to oversee and manage the water source and environment to provide clean, uncontaminated drinking water. Increasing the severity of punishments would assist in preventing unlawful sewage releases while enhancing oversight and monitoring would assure the efficacy of handling pollution situations. Thus, respondents saw supervision, monitoring, and resource management as effective water pollution control techniques.

### 3.4. Comparative Analysis of All Five Tehsils of Faisalabad

Based on survey findings, inhabitants of five tehsils have distinct levels of student knowledge about drinking water concerns. These distinctions are also shown in Tables 2 and 3. The Samundri tehsil has less contaminated underground water, and inhabitants believe that tap water is safe to drink, with the highest tap water average (44.4%) compared to all other tehsils. Faisalabad Sadar is a more congested area with massive industry and contaminated underground water. Due to these factors, inhabitants of Faisalabad Sadar believe their drinking water is insufficiently safe and must be more cautious and aggressive than residents of other tehsils. So most residents (42.1%) of Faisalabad Sadar use barreled or bottled water; however, only 25% of Chak Jhumra residents use barreled or bottled water; spring water is highly used in Chak Jhumra at 14.3% (2.2% overall). Regarding public perceptions of drinking water safety, 46.4% of inhabitants of Chak Jhumra have more trust in drinking water quality of local supply than Jaranwala, 19% lowest, and the overall average of the Fasislabad district is 28.5%. And, concerning the level of satisfaction with their drinking water quality, people of Faisalabad Sadar were less happy (10.5%), while residents of Samundri were more satisfied with drinking water quality (33.3%). Faisalabad Sadar residents pay more attention to the quality of the local water supply pollution events (47.4%), and Jaranawala residents pay less attention. Faisalabad Sadar inhabitants had a lower degree of confidence in the safety of drinking water but were more concerned about the safety of drinking water. These results suggested that Faisalabad Sadar inhabitants are more likely to see their drinking water as less safe and are less happy with it. However, there are no major variations between all five tehsils in terms of public understanding of the primary variables affecting the quality of drinking water and the efforts taken to address issues with tap water.

### 3.5. Factors Influencing Student Satisfaction with Safe Drinking Water

The multinomial logistic regression (MLR) model is applied to assess the relations between the level of public awareness of the quality of drinking water and its affecting foundations. The results are shown in Table 4: according to panel 1 of Table 4, there were no significant associations between age, level of education, and satisfaction with drinking water quality. Nevertheless, gender and residential area played a statistically significant role in distinguishing between three groups of respondents: those who were very satisfied and dissatisfied with the quality of their drinking water and those who were between satisfied and dissatisfied. Panel 2 of Table 4 shows the association between public attention to local water quality and its affecting variables. Age and the residential area had no significant link with water quality awareness. However, gender and education were statistically significant in distinguishing the three groups. Those with higher degrees of education exhibited greater levels of understanding than those with lower levels of education. Panel 3 of Table 4 depicts the results of public trust in the safety of drinking water. Age and education have no significant impact on the confidence level. However, the gender and tehsil of residence have an important negative relationship with the level of confidence about water safety.

### 3.6. Influencing Factors of Student Awareness regarding Drinking Water Contamination Accidents

A multinomial logistic regression model investigated the association between student knowledge of drinking water contamination accidents and its affecting variables. Panel 1 of Table 5 indicates that age and residence status did not significantly influence drinking water pollution awareness. However, gender and degree of education did play statistically significant roles in differing awareness levels across groups. Those with a greater level of education were more aware of contamination incidents than those with a lower level of education. Panel 2 of Table 5 shows that age, gender, and area of residence do not have any significant relationship with the suggestion to reduce water contamination accidents. But the level of education has an important relationship with strengthening the supervision and propaganda.

Keeping customers informed about the quality of their drinking water is crucial to maintaining public health [1, 31, 34]. Thus, public awareness affecting elements might potentially reflect drinking water safety and pollution incidents and offer decision-makers vital information. If we disregard the fact that people of all ages and both sexes have access to information about drinking water emergencies through television, newspapers, and the Internet, age and gender did not play a significant role in the degree of public satisfaction with water quality and public perception of water pollution accidents. The factors influencing students' views of drinking water might vary considerably among populations. For instance, in terms of general knowledge of drinking water pollution incidents, males were more aware than women; yet there were no significant differences between men and women in their occasional interest in contamination occurrences. Similar to prior environmental research [16], respondents' views and behavior were heavily influenced by their level of education. Respondents with a higher education level were more aware of local water quality and water pollution incidents than those with a lower education level.

## 4. Conclusions

The research examined the student's knowledge of drinking water safety and pollution accidents and the link between awareness of these concerns and its primary influencing elements. Specifically, a questionnaire survey was conducted in the five tehsils of the Faisalabad district, Pakistan. We observed that respondents with some knowledge about their water quality are more confident in their drinking water and give more support for water safety and pollution avoidance. The majority of respondents in this research believe they have a good level of knowledge about drinking water quality and safety and pollution incidents. Approximately 66% of respondents were concerned about local water quality (special attention 36.0% and comparatively close attention 30.2%). Only 22% of respondents were very happy with the quality of their drinking water, while 62% were somewhat satisfied. Education level and health monitoring of drinking water quality might impact public knowledge of drinking water safety and contamination incidents. The study findings support the implementation of proper monitoring and public policies to ensure integrated and sustainable water development and minimize health risks in the study area. It will influence the decision-making process for enhancing drinking water quality monitoring to assure its safety. It also instructs them to boost student awareness of drinking water quality, strengthen education, expand understanding of drinking water safety, and enhance emergency response for drinking water contamination incidents. Using local television and print media, it is possible to boost public satisfaction by highlighting the significance of the local government's yearly report on the quality of drinking water. This research was conducted in one of Pakistan's most significant agricultural and industrial areas, Faisalabad. The findings may inform policy for other metropolitan areas of the same kind. For future research, it is proposed that a greater region be covered and information concerning illnesses caused by polluted water be added if possible.

## Figures and Tables

**Table 1:** Demographic information of participants.

	Faisalabad (total)		Chak Jhumra		Faisalabad Sadar		Jaranwala		Samundri		Tandlianwala	
	*N*	%Age (%)	*N*	%Age (%)	*N*	%Age (%)	*N*	%Age (%)	*N*	%Age (%)	*N*	%Age (%)
*Age*												
<20	65	15.8	11	13.1	9	15.8	19	17.6	8	14.8	18	16.7
20–34	115	28.0	22	26.2	16	28.1	31	28.7	17	31.5	29	26.9
35–50	173	42.1	37	44.1	24	42.1	43	39.8	22	40.7	47	43.5
>50	58	14.1	14	16.7	8	14.0	15	13.9	7	13.0	14	13.0

*Gender*												
Male	216	52.9	45	53.6	33	57.9	54	50.0	33	61.1	51	48.6
Female	192	47.1	39	46.4	24	42.1	54	50.0	21	38.9	54	51.4

*Education*												
Bachelor's	330	82.7	60	74.1	48	84.2	87	82.9	42	82.4	93	88.6
Master's or above	45	11.3	15	18.5	9	15.8	15	14.3	3	5.9	3	2.9

Source: Author survey.

**Table 2:** Student awareness of drinking water safety (statistical results).

	Faisalabad (total)		Chak Jhumra		Faisalabad Sadar		Jaranwala		Samundri		Tandlianwala	
	*N*	%Age	*N*	%Age	*N*	%Age	*N*	%Age	*N*	%Age	*N*	%Age
*Panel 1: Main source of drinking water*												
Tap water	153	37.2	30	35.7	21	36.8	42	38.9	24	44.4	36	33.3
Barreled or bottled water	138	33.6	21	25.0	24	42.1	33	30.6	21	38.9	39	36.1
Well water	57	13.9	0	0.0	0	0.0	18	16.7	6	11.1	15	13.9
Spring water	9	2.2	12	14.3	6	10.5	3	2.8	3	5.6	3	2.8
Others	54	13.1	21	25.0	6	10.5	12	11.1	0	0.0	15	13.9

*Panel 2: Attention to local drinking water quality*												
Special attention	147	36.0	27	32.1	27	47.4	42	38.9	12	23.5	39	36.1
Comparatively high attention	123	30.2	30	35.7	12	21.1	27	25.0	21	41.2	33	30.6
Not concerned	114	27.9	24	28.6	15	26.3	30	27.8	12	23.5	33	30.6
No answer	24	5.9	3	3.6	3	5.3	9	8.3	6	11.8	3	2.8

*Panel 3: Satisfaction level with drinking water quality*												
Very satisfied	90	21.9	18	21.4	6	10.5	15	13.9	18	33.3	33	30.6
Relatively satisfied	255	62.0	54	64.3	45	79.0	60	55.6	33	61.1	63	58.3
Dissatisfied	45	11.0	9	10.7	3	5.3	21	19.4	3	5.6	9	8.3
No answer	21	5.1	3	3.6	3	5.3	12	11.1	0	0.0	3	2.8

*Panel 4: Trust level in the safety of drinking water*												
Confident	117	28.5	39	46.4	18	31.6	21	19.4	15	27.8	24	22.2
Relatively confident	114	27.7	21	25.0	18	31.6	21	19.4	21	38.9	33	30.6
Somewhat worried	138	33.6	21	25.0	9	15.8	51	47.2	0	0.0	42	38.9
Extremely worried	30	7.3	3	3.6	9	15.8	9	8.3	15	27.8	9	8.3
No answer	12	2.9	0	0.0	3	5.3	6	5.6	3	5.6	0	0.0

*Panel 5: Awareness of problems with tap water quality*												
Never had problems	99	24.1	24	28.6	12	21.1	21	19.4	18	33.3	24	22.2
Had problems once or twice a year	144	35.0	24	28.6	24	42.1	27	25.0	24	44.4	45	41.7
Had problems frequently	138	33.6	27	32.1	18	31.6	48	44.4	6	11.1	39	36.1
No answer	30	7.3	9	10.7	3	5.3	12	11.1	6	11.1	0	0.0

*Panel 6: Measures taken to solve problems that arise with tap water*												
Solve problems by themselves	195	47.5	57	67.9	33	57.9	30	27.8	33	61.1	42	38.9
Help by local water utility	102	24.8	6	7.1	0	0.0	39	36.1	15	27.8	42	38.9
Complain to the local department of health	36	8.8	3	3.6	9	15.8	12	11.1	3	5.6	9	8.3
Help by the residential property maintenance staff	39	9.5	12	14.3	9	15.8	12	11.1	0	0.0	6	5.6
Call the local government telephone hotline for help	39	9.5	6	7.1	6	10.5	15	13.9	3	5.6	9	8.3

**Table 3:** Student awareness of drinking water contamination accidents (statistical results).

	Faisalabad (total)		Chak Jhumra		Faisalabad Sadar		Jaranwala		Samundri		Tandlianwala	
	*N*	%Age	*N*	%Age	*N*	%Age	*N*	%Age	*N*	%Age	*N*	%Age
*Panel 1: Attention to the water pollution events*												
Pay special attention	141	34.3	33	39.3	27	47.4	27	25.0	21	38.9	33	30.6
Follow in free time	180	43.8	33	39.3	27	47.4	45	41.7	24	44.4	51	47.2
Not concerned	72	17.5	9	10.7	3	5.3	33	30.6	6	11.1	21	19.4
No answer	18	4.4	9	10.7	0	0.0	3	2.8	3	5.6	3	2.8

*Panel 2: Attention-seeking water pollution events*												
Damage to human health	345	83.9	72	85.7	54	94.7	75	69.5	42	77.8	102	94.4
Influence scales	18	4.4	3	3.6	3	5.3	6	5.6	6	11.1	0	0.0
Cause of accident	27	6.6	9	10.7	0	0.0	9	8.3	3	5.6	6	5.6
Accident information publication	12	2.9	0	0.0	0	0.0	12	11.1	0	0.0	0	0.0
Accident treatment procedures	9	2.2	0	0.0	0	0.0	6	5.6	3	5.6	0	0.0

*Panel 3: Emergency response provider in water contamination accidents*												
Health department	147	36.0	36	42.9	18	31.6	30	27.8	21	38.9	42	40.0
Environmental protection department	33	8.1	6	7.1	3	5.3	12	11.1	9	16.7	3	2.9
Water resources department	183	44.9	42	50.0	27	47.4	48	44.4	15	27.8	51	48.6
Propaganda department	18	4.4	0	0.0	3	5.3	6	5.6	6	11.1	3	2.9
Housing and urban, rural development department	27	6.6	0	0.0	6	10.5	12	11.1	3	5.6	6	5.7

*Panel 4: Things to due to reducing pollution emergencies*												
Strengthening supervision	108	26.5	24	28.6	12	21.1	27	25.0	21	38.9	24	22.9
Resource management	174	42.7	33	39.3	33	57.9	33	30.6	24	44.4	51	48.6
Propaganda for protecting the knowledge	39	9.6	12	14.3	3	5.3	6	5.6	3	5.6	15	14.3
Increasing the intensity of the punishment	87	21.3	15	17.9	9	15.8	42	38.9	6	11.1	15	14.3

**Table 4:** Influencing factors of public awareness about water safety (multinomial logistic regression results).

Variables	Panel 1: Satisfaction level with drinking water quality			Panel 2: Public attention to local drinking water quality			Panel 3: Trust level in the safety of drinking water			
	Very satisfied	Dissatisfied	No answer	High attention	Not concerned	No answer	Confident	Relatively confident	Extremely worried	No answer
Age	0.0434	−0.149	0.0737	0.0790	0.138	0.0336	0.0105	0.0249	0.0350	−0.334
	(0.138)	(0.176)	(0.271)	(0.140)	(0.140)	(0.241)	(0.140)	(0.141)	(0.221)	(0.385)

Gender	0.480^*∗*^	0.690^*∗∗*^	−0.395	0.799^*∗∗∗*^	0.869^*∗∗∗*^	−0.499	−0.499^*∗*^	−0.468^*∗*^	−0.133	14.89
	(0.253)	(0.331)	(0.524)	(0.260)	(0.261)	(0.503)	(0.259)	(0.261)	(0.405)	(653.0)

Education	0.192	0.116	1.489^*∗∗∗*^	−0.717^*∗∗∗*^	0.603^*∗*^	0.391	−0.140	0.0583	−0.0456	−0.727^*∗∗*^
	(0.208)	(0.262)	(0.540)	(0.248)	(0.315)	(0.549)	(0.208)	(0.225)	(0.329)	(0.314)

Tehsil	0.192^*∗∗*^	−0.0153	0.0233	0.0187	0.0867	0.0757	−0.293^*∗∗∗*^	−0.00803	−0.103	−0.200
	(0.0891)	(0.113)	(0.183)	(0.0892)	(0.0911)	(0.158)	(0.0911)	(0.0923)	(0.142)	(0.239)

Constant	−2.785^*∗∗∗*^	−2.112^*∗*^	−8.936^*∗∗∗*^	1.999^*∗*^	−3.754^*∗∗∗*^	−3.559	1.507	−0.272	−1.011	−12.82
	(1.034)	(1.280)	(2.626)	(1.152)	(1.443)	(2.514)	(1.031)	(1.104)	(1.633)	(653.0)
Observations	399	399	399	396	396	396	399	399	399	399
Pseudo-r-squared	28%			51%			34%			
Chi-square	22.619^*∗∗*^			51.057^*∗∗*^			37.142^*∗∗∗*^			
Akaike crit. (AIC)	815.160			972.506			1,088.314			
Ref. category	Relatively satisfied			Special attention			Somewhat worried			

Standard errors are provided in parentheses. ^*∗∗∗*^*p* < 0.01, ^*∗∗*^*p* < 0.05, and ^*∗*^*p* < 0.1.

**Table 5:** Influencing factors of student awareness about water contamination accidents (multinomial logistic regression results).

Variables	Panel 1: Attention to the water pollution events			Panel 2: Things to due to reducing pollution emergencies		
	Pay special attention	Not concerned	No answer	Strengthening supervision	Propaganda for protecting the knowledge	Increasing the intensity of the punishment
Age	−0.00487	−0.0130	0.222	−0.0738	0.0638	0.0149
	(0.126)	(0.154)	(0.354)	(0.139)	(0.199)	(0.145)

Gender	−0.516^*∗∗*^	0.407	−1.419^*∗*^	0.286	−0.346	0.128
	(0.234)	(0.290)	(0.737)	(0.256)	(0.369)	(0.267)

Education	0.105	0.452^*∗*^	−0.607^*∗*^	−0.774^*∗∗∗*^	−0.685^*∗∗*^	0.477
	(0.188)	(0.272)	(0.358)	(0.237)	(0.294)	(0.313)

Tehsil	−0.0895	0.0893	−0.662^*∗∗∗*^	−0.0819	−0.0353	−0.0596
	(0.0812)	(0.101)	(0.244)	(0.0889)	(0.125)	(0.0945)

Constant	−0.210	−3.263^*∗∗*^	1.262	2.909^*∗∗∗*^	1.373	−2.523^*∗*^
	(0.929)	(1.307)	(1.913)	(1.121)	(1.441)	(1.442)
Observations	399	399	399	396	396	396
Pseudo-r-squared	40%			30%		
Chi-square	38.284^*∗∗∗*^			30.13^*∗∗∗*^		
Akaike crit. (AIC)	956.937			1,012.031		
Ref. category	Follow in free time			Resource management		

Standard errors are provided in parentheses. ^*∗∗∗*^*p* < 0.01, ^*∗∗*^*p* < 0.05, and ^*∗*^*p* < 0.1.

## Data Availability

The data sets used and/or analyzed during the current study can be obtained from the corresponding author upon reasonable request.

## Supplementary Materials

Supplementary Materials The questionnaire used for the survey has been attached as supplementary material.
