# Supplementary material for: Awareness Level of Business Students regarding Drinking Water Safety and Associated Adulteration Accidents: A Multinomial Logistic Regression Approach
Source: J Environ Public Health. 2022 Aug 29;2022:7492409. doi: 10.1155/2022/7492409 (PMC9444455; doi:10.1155/2022/7492409)
Supplement: Supplementary Materials — The questionnaire used for the survey has been attached as supplementary material. [file 7492409.f1.docx]

**Supplementary material**

**Household Survey for assessing the Public Awareness of Drinking Water Safety and Contamination Accidents: A Case study from Faisalabad (Pakistan)**

**Part A**

Q1 Name: optional___________________

Q2: Age A) 18-29 B) 30-39 C) 40-49 C) 50 or above (A=1, B=2, C=3,D=4)

Q3: Sex A) Male B) Female (A=0, B=1)

Q4: Education A) Primary School or Below B) High School C) Bachelor D) Master (A=1, B=2, C=3, D=4)

Q5: A) Chak Jhumra B) Faisalabad Sadar C) Jaranwala D) Samundri E) Tandlianwala

**B: Public Awareness of Drinking water Safety**

**B01**: What kind of water do you use as the main source of your drinking water?

1. Tap water 2. Barreled or bottled water 3. Well water 4. Spring water 5. Others

**B02**: Do you pay attention to local drinking water quality?

1. Special attention 2. Comparatively high attention 3. No concerned 4. No answer

**B03:** Are you satisfied with your drinking water quality?

1. Very satisfied 2. Relatively satisfied 3. Dissatisfied 4. No answer

**B04:** Do you trust the safety of your drinking water?

1. Confident 2. Relatively confident 3. Somewhat worried 4. Extremely worried 5. No answer

**B05:** Do you have any problems with your tap water quality?

1. Never had problems 2. Had problems once or twice a year 3. Had problems frequently 4. No answer

**B06:** When you have problems with your drinking water (such as water quality abnormal, pipeline damage, faucet water leakage, etc.), how do you solve these problems?

1. Solve problems by themselves
2. Help by local water utility
3. Complain to the local department of health
4. help by the residential property maintenance staff
5. 5. call the local government telephone hotline for help

**C: Public Awareness about water contamination accidents**

**C01:** Do you pay attention to the water pollution events reported on TV or in the newspaper?

1. Pay special attention
2. Follow in free time
3. Not concerned
4. No answer

**C02:** What kind of water pollution events do you pay attention to?

1. Damage to human health
2. Influence scales
3. Cause of accident
4. Accident information publication
5. Accident treatment procedures

**C03:** Who are the main emergency response providers during drinking water contamination accidents?

1. Health department
2. Environmental protection department
3. Water resources department
4. Propaganda department
5. Housing and urban, rural development department

**C04:** What do you think should be done to reduce pollution emergencies?

1. Strengthening supervision
2. Resource Management
3. Propaganda for protecting the knowledge
4. Increasing the intensity of the punishment.
